# Supplementary material for: Bayesian refinement of protein structures and ensembles against SAXS data using molecular dynamics
Source: PLoS Comput Biol. 2017 Oct 18;13(10):e1005800. doi: 10.1371/journal.pcbi.1005800 (PMC5662244; doi:10.1371/journal.pcbi.1005800)
Supplement: S1 Archive — (BZ2) [file pcbi.1005800.s011.tar.bz2 › gromacs_4.62_bayesian_saxs_refi/share/html/online/g96.html]

g96 file format


## g96 file format

|  |  |
| --- | --- |
| Main Table of Contents | **VERSION 4.6** |
| GROMACS homepage | **Sat 19 Jan 2013** |

---

A file with the g96 extension can be a GROMOS-96 initial/final
configuration file or a coordinate trajectory file or a combination of both.
The file is fixed format, all floats are written as 15.9 (files can get huge).
GROMACS supports the following data blocks in the given order:

- Header block:
  - TITLE (mandatory)
  - Frame blocks:
    - TIMESTEP (optional)- POSITION/POSITIONRED (mandatory)- VELOCITY/VELOCITYRED (optional)- BOX (optional)

See the GROMOS-96 manual for a complete description of the blocks.

Note that all GROMACS programs can read compressed or g-zipped files.

---

http://www.gromacs.org
